# Early Outcomes of Dorsal Onlay Buccal Mucosal Graft Urethroplasty for Female Urethral Stricture Disease: A Single-Center Experience

**DOI:** 10.3390/medicina62101976

**Published:** 2026-10-13

**Authors:** Hilmi Sarı, Murat Can Karaburun, Arif Huseynli, Samet Kipik, Fatih Yalçınkaya

**Affiliations:** Ankara Etlik City Hospital, Ankara 06170, Turkey; dr.huseynliarif@gmail.com (A.H.); sametkipik@gmail.com (S.K.); nykaya2@yahoo.com (F.Y.)

**Keywords:** female, urethral stricture, urethroplasty, buccal mucosal graft, dorsal, onlay

## Abstract

*Background and Objectives*: To evaluate the surgical, functional, and perioperative outcomes of Dorsal Onlay Buccal Mucosal Graft Urethroplasty (DO-BMGU) in women with Female urethral stricture (FUS) disease. *Materials and Methods*: A retrospective analysis was performed including women who underwent DO-BMGU for FUS at a single tertiary referral center between January 2018 and October 2025. The primary outcome was freedom from reintervention for symptomatic recurrence. Secondary outcomes included perioperative outcomes, complications, and changes in maximum urinary flow rate (Qmax) and postvoid residual urine volume (PVR). *Results***:** Twenty women with a mean age of 52.9 ± 14.7 years were included. The median stricture length was 2 cm (IQR 2–3), and all patients had undergone previous urethral dilatation. Median operative time was 65 min (IQR 60–80), with a median hospital stay of 3 days (IQR 3–4). During a median follow-up of 12 months (IQR 12–24), 18 patients (90%) remained free from reintervention for symptomatic recurrence. Two patients developed recurrent symptomatic narrowing and were successfully managed with urethral dilatation. At the 3-month objective functional assessment, mean Qmax improved significantly from 6.1 ± 2.3 to 20.4 ± 5.8 mL/s (*p* < 0.001), while median PVR decreased from 125 mL (IQR 94.5–155) to 10 mL (IQR 10–40) (*p* < 0.001). No patient experienced major bleeding or urethrovaginal fistula. One patient (5%) developed mild de novo stress urinary incontinence, and postoperative complications were generally minor and managed conservatively. *Conclusions*: DO-BMGU was associated with favorable early objective functional outcomes and a high rate of freedom from reintervention for symptomatic recurrence, with few clinically significant complications in this small cohort. Larger prospective studies with longer follow-up are warranted to confirm the long-term durability of this technique.

## 1. Introduction

Female urethral stricture (FUS) is an uncommon cause of lower urinary tract symptoms (LUTS) and bladder outlet obstruction (BOO) in women. BOO is diagnosed in fewer than 10% of women presenting with LUTS, and FUS accounts for approximately 4–18% of these cases [1,2]. Because its symptoms are often nonspecific and may overlap with other causes of female voiding dysfunction, FUS remains a challenging and potentially underdiagnosed condition. Diagnosis is generally based on a combination of clinical assessment, uroflowmetry, postvoid residual urine measurement, urodynamic findings, voiding cystourethrography, and urethrocystoscopy [2].

Urethral dilation has historically been the most commonly used treatment for FUS. However, recurrence and the need for repeated dilation or self-dilation, together with decreasing success after repeated procedures, have increased interest in definitive urethral reconstruction [3,4]. Both dorsal and ventral urethroplasty techniques have demonstrated favorable outcomes and are increasingly used in contemporary reconstructive practice. Furthermore, urethroplasty has also been successfully performed in patients without previous urethral dilation [5,6]. Several graft materials and surgical approaches have been described, with both vaginal and buccal mucosal grafts providing favorable outcomes [7].

Among these options, buccal mucosa is widely used in urethral reconstruction, and dorsal onlay placement provides a reconstructive approach that avoids a ventral urethral incision. Nevertheless, FUS remains uncommon, and clinical experience with individual reconstructive techniques is consequently based on relatively limited patient series. Further reporting of technique-specific functional outcomes, recurrence, and procedure-related morbidity may therefore contribute to the available evidence on female urethral reconstruction. The present study aimed to evaluate the early objective functional outcomes, freedom from reintervention for symptomatic recurrence, perioperative outcomes, and complications of dorsal onlay buccal mucosal graft urethroplasty (DO-BMGU) in women with FUS.

## 2. Material and Methods

### 2.1. Study Design and Patient Population

This study was conducted in accordance with the Declaration of Helsinki and its later revisions and received approval from the local ethics committee. Institutional review board approval for the retrospective analysis of existing clinical data was obtained on 27 January 2026 (approval no. AESH-BADEK2-2026-083). We retrospectively analyzed a database of women aged > 18 years who underwent dorsal onlay BMG urethroplasty for FUS at a single tertiary center between January 2018 and October 2025. All patients had provided written preoperative informed consent permitting the use of their clinical data for scientific research. Exclusion criteria were lack of consent, neurological disorders, primary bladder neck contracture, previous urethroplasty, or ancillary surgery (prolapse correction, urethral diverticulum or caruncle excision, or Martius flap interposition). Given the rarity of FUS and the retrospective design of the study, no formal sample-size calculation was performed; all eligible patients undergoing DO-BMGU during the study period were included. The primary clinical outcome was freedom from reintervention for symptomatic recurrence. Secondary outcomes included complications and changes in maximum urinary flow rate (Qmax) and postvoid residual volume (PVR).

### 2.2. Preoperative Evaluation and Diagnostic Criteria

During the study period, 24 women underwent urethral reconstructive surgery at our institution. All eligible patients who underwent DO-BMGU for female urethral stricture during this period were included in the present study (*n* = 20). Four patients who underwent reconstructive procedures for other urethral pathologies were excluded: urethral repair following mesh erosion (*n* = 1), urethrovaginal fistula repair with a Martius flap (*n* = 1), periurethral cyst excision with concomitant urethral repair (*n* = 1), and caruncle excision with distal urethral repair (*n* = 1). Patients considered for DO-BMGU had recurrent FUS following previous endoscopic treatment. The selection of DO-BMGU as the reconstructive approach reflected surgeon preference and institutional practice during the study period.

All patients underwent clinical history assessment, physical examination, urinalysis, urine culture, uroflowmetry, and postvoid residual urine assessment. Diagnostic criteria included difficulty calibrating the urethra with a 12-Fr catheter, Qmax < 12 mL/s, and anatomical narrowing confirmed by urethroscopy. A voided volume of ≥150 mL was generally targeted for uroflowmetry; one 3-month measurement obtained at 109 mL was retained in the analysis. Urethroscopy was performed in all 20 patients.

### 2.3. Postoperative Follow-Up and Outcome Assessment

Postoperative follow-up was scheduled at 1, 3, 6, and 12 months and annually thereafter. Uroflowmetry and PVR were systematically available at 3 months but not consistently at later visits; subsequent follow-up therefore primarily assessed recurrent or worsening lower urinary tract symptoms and the need for further intervention. Symptoms were assessed clinically, without a validated questionnaire. When recurrence was suspected, further evaluation included uroflowmetry, PVR assessment and/or urethroscopy, as clinically indicated. The primary clinical outcome was freedom from reintervention for symptomatic recurrence, with reintervention defined as any additional procedure, including urethral dilatation, for recurrent symptomatic urethral narrowing. Follow-up duration was calculated from surgery to the last documented clinical visit. Patients who did not return thereafter were considered lost to further follow-up, and no assumptions were made regarding recurrence after the last available assessment. Postoperative complications occurring within 30 days after surgery were recorded and classified according to the Clavien–Dindo system, where applicable.

### 2.4. Surgical Technique

All procedures were performed under general anesthesia in the lithotomy position by three senior urologists (HS, AH, and FY) experienced in urethral reconstruction and BMG urethroplasty. The same standardized surgical technique was used throughout the study period, without major modifications in the principal surgical steps. Patients received 2 g of intravenous cefazolin at induction. Urethrocystoscopy with a 6.5-Fr ureterorenoscope was performed to assess stricture location, length, and proximity to the bladder neck. An inverted-U suprameatal dorsal incision was made, and a plane was developed between the urethra and clitoral cavernosal bed. A dorsal urethrotomy was extended 1 cm proximal to the stricture until satisfactory caliber was achieved. Stricture length was measured intraoperatively and the BMG tailored accordingly. The graft was secured proximally with two 4-0 Vicryl apical sutures, its edges were sutured to the urethral mucosa, and the graft was quilted to the clitoral bed with interrupted absorbable sutures. A silicone urethral catheter was maintained for 21 days according to our institutional reconstructive protocol.

### 2.5. Buccal Mucosal Graft Harvest

The BMG was harvested by a senior urologist using a Kilner–Doughty oral retractor. Stricture length was measured intraoperatively using a sterile surgical ruler, and the BMG was planned to a length corresponding to the measured stricture length. After identification of the Stensen duct, the required graft dimensions were marked on the buccal mucosa using a sterile surgical marking pen. Graft width was tailored intraoperatively to the urethral defect. Bupivacaine with epinephrine was injected along the graft margins to facilitate hemostasis and dissection. The graft was dissected from the underlying muscular layer and harvested, and the donor site was sutured closed.

### 2.6. Statistical Analyses

Statistical analyses were performed using IBM SPSS Statistics for Windows, version 20.0 (IBM Corp. Armonk, NY, USA). Normality was assessed using the Shapiro–Wilk test. Normally distributed variables are presented as mean ± standard deviation (SD), non-normally distributed variables as median and interquartile range (IQR), and categorical variables as numbers and percentages. Paired continuous variables were compared using the paired-samples *t*-test or Wilcoxon signed-rank test, as appropriate. A two-sided *p* < 0.05 was considered statistically significant. Freedom from reintervention for symptomatic recurrence was reported with an exact 95% confidence interval using the Clopper–Pearson method.

## 3. Results

During the study period, 24 women with FUS were assessed for eligibility. Four patients who underwent reconstructive procedures other than DO-BMGU were excluded, leaving 20 patients who underwent DO-BMG and were included in the final analysis. The mean age was 52.9 ± 14.7 years, and the mean body mass index (BMI) was 28.1 ± 5.0 kg/m^2^. According to the American Society of Anesthesiologists (ASA) classification, 12 patients (60%) were classified as ASA II, while four patients (20%) each were classified as ASA I and ASA III. Only two patients (10%) were active smokers, and smoking status was unavailable for nine patients (45%) (Table 1). Idiopathic stricture was the most common etiology, accounting for 55% (*n* = 11) of cases, followed by iatrogenic strictures (30%, *n* = 6). Infectious/inflammatory, lichen sclerosus, and traumatic etiologies each accounted for one patient (5%). The patient with lichen sclerosus underwent BMG urethroplasty and remained free from symptomatic recurrence and reintervention during follow-up.

The majority of strictures were located in the midurethra (45%, *n* = 9), followed by the distal urethra (35%, *n* = 7), whereas proximal and panurethral strictures each accounted for two cases (10%). The median stricture length was 2 cm (IQR, 2–3). All patients had undergone at least one previous urethral dilatation, with a median of 2.5 (IQR, 1.8–4.0) dilatations, and three patients (15%) had a history of internal urethrotomy. Preoperatively, the mean maximum urinary flow rate (Qmax) was 6.1 ± 2.3 mL/s, and the median postvoid residual (PVR) volume was 125 mL (IQR, 94.5–155) (Table 1).

The median operative time was 65 min (IQR, 60–80). Patients were hospitalized for a median of 3 days (IQR, 3–4), and the Foley catheter was removed at a median of 21 days (IQR, 21–21) postoperatively. The median clinical follow-up was 12 months (IQR, 12–24; range, 6–60 months). Sixteen patients (80%) had at least 12 months of follow-up, six (30%) had at least 24 months, five (25%) had more than 24 months, four (20%) had at least 36 months, and one (5%) had at least 48 months of follow-up.

The four patients with less than 12 months of follow-up had undergone surgery relatively recently and had not yet reached the 12-month follow-up time point. Five patients were lost to further follow-up after their last documented visits at 27, 36, 36, 42, and 60 months, respectively. During the available clinical follow-up, 18 of 20 patients (90%; 95% CI, 68.3–98.8%) remained free from reintervention for symptomatic recurrence (Table 2). Two patients developed worsening lower urinary tract symptoms with recurrent narrowing at 6 and 9 months, respectively, and both were managed with urethral dilatation.

At the 3-month objective functional assessment, paired Qmax and PVR measurements were available for all 20 patients. Mean Qmax increased significantly from 6.1 ± 2.3 mL/s preoperatively to 20.4 ± 5.8 mL/s, corresponding to a mean increase of 14.30 mL/s (95% CI, 11.38–17.22; *p* < 0.001). Likewise, median PVR decreased significantly from 125 mL (IQR, 94.5–155) to 10 mL (IQR, 10–40) (*p* < 0.001). Objective Qmax and PVR measurements were not consistently available for the entire cohort at subsequent follow-up visits and were therefore not analyzed longitudinally.

Postoperative complications were assessed within 30 days after surgery and classified according to the Clavien–Dindo system where applicable. Overall, 5 of 20 patients (25%) experienced at least one postoperative complication or adverse event, with multiple events occurring in some patients. Four patients (20%) developed catheter-associated urinary tract infections, and one of these patients also developed a superficial urethral/perineal surgical-site infection. These infections required antibiotic therapy and were classified as Clavien–Dindo grade II. No Clavien–Dindo grade III or higher complications occurred. One patient (5%) developed de novo mild stress urinary incontinence, diagnosed based on clinical history and gynecological examination. Pelvic floor muscle (Kegel) exercises were recommended without additional medical therapy, and this event was reported separately as a functional adverse outcome.

Oral donor-site morbidity was transient and self-limiting. Reported events included postoperative bleeding in one patient (5%), local inflammatory changes at the donor site in two (10%), cheek swelling in one (5%), hypergranulation tissue in one (5%), difficulty with mouth opening and chewing lasting > 10 days in three (15%), and articulation difficulty lasting > 10 days in two patients (10%) (Table 3). The two patients with local donor-site inflammatory changes also experienced transient difficulty with mouth opening/chewing and articulation. All mouth-opening/chewing and articulation difficulties resolved completely within 1 month with conservative management. No oral donor-site morbidity required surgical intervention.

## 4. Discussion

In this descriptive study, we aimed to report the clinical, early objective voiding, and perioperative outcomes of DO-BMGU for FUS. During a median clinical follow-up of 12 months, 90% of patients remained free from reintervention for symptomatic recurrence. Assessment of objective voiding parameters at 3 months demonstrated significant improvement in Qmax and PVR. This small single-center series adds a detailed description of early outcomes after DO-BMGU in women with recurrent FUS following previous endoscopic treatment, including perioperative and donor-site morbidity. Given the small cohort and relatively short follow-up, these findings should be interpreted as descriptive early outcomes rather than evidence of long-term durability.

Over the last several years, there has been a substantial rise in consciousness of FUS, both in terms of diagnosis and treatment methods. FUS presents a challenging diagnostic conundrum, with several variables potentially influencing its development. In the present study, iatrogenic and idiopathic factors were the most common. In contemporary research, iatrogenic and idiopathic factors mostly represent the majority of causes [8]. The female urethra is short, and the majority of strictures documented in the literature are located in the distal-mid region [6,8]. In our study, the reporting of 80% distal or mid urethral strictures is also consistent with the literature. When it comes to treatment, urethral dilation has been the primary component for a long period of time. Urethral dilation is still recommended by the European Association of Urology (EAU) guidelines as a first-line therapy and at the first recurrence, despite the fact that the degree of recommendation is weak [9]. Unfortunately, success remains low with this treatment method. Conversely, the body of literature concerning reconstructive treatment techniques is progressively gaining robustness. Several techniques using grafts and flaps have been delineated. In these methods, grafts or flaps can be applied as onlays or inlays utilizing either a dorsal or ventral approach [4]. Consequently, urethroplasty is performed after unsuccessful dilatations and as an initial intervention [10]. A series of over 200 patients who underwent dorsal onlay labial mucosal graft urethroplasty was recently published by Jena et al. In this study, 88% of the 204 patients had no prior history of dilation and overall success rate was 93.5% at 2 year median follow-up. The authors emphasize that urethroplasty could be performed at an earlier stage, which would reduce periurethral fibrosis and increase the success rate of urethroplasty [6]. While Jena et al. promoted primary urethroplasty, it is now standard in many clinics to consider urethroplasty after dilation failure in line with current guidelines. Another substantial cohort, including 165 female urethroplasty patients, has been published by the “Female Reconstruction Group” associated with the “European Association of Urology Section of Genitourinary Reconstructive Surgeons” [8]. In this study Waterloos et al. compared ventral onlay flap, ventral onlay graft and dorsal onlay graft urethroplasty techniques reported 73%, 86% and 80% 2-year stricture free survival rates, respectively [9].

Contemporary retrospective studies, randomized controlled trials, and meta-analyses have reported favorable outcomes with dorsal onlay urethroplasty [10,11,12,13,14]. However, heterogeneity in patient selection, outcome definitions, and follow-up limits definitive comparisons between reconstructive techniques. Randomized controlled trials remain limited because of the rarity of the disease. In the first RCT regarding this subject, Katiyar et al. compared dorsal onlay and ventral onlay urethroplasties, revealing similar high success rates in both groups [14]. No notable intraoperative or postoperative complications were seen in any patient. The dorsal technique did not result in stress incontinence or bleeding, which are commonly feared complications. The other RCT by Mandal et al. compared ventral inlay and dorsal onlay techniques. The authors reported a 95% success rate in both groups at 1-year follow-up. Median blood loss was 15 mL for DO-BMGU versus 10 mL for VI-BMGU (*p* = 0.012), and median operative time was 44.5 versus 30 min, respectively (*p* < 0.001), both favoring the ventral inlay approach [12]. Using freedom from reintervention for symptomatic recurrence as the principal clinical outcome, 90% of patients remained intervention-free during follow-up. This finding is broadly consistent with the high success rates reported in previous female urethroplasty series.

In a recent study by Karaburun et al., redo dorsal onlay BMG urethroplasty performed in female patients with prior failed ventral urethroplasty, yielding a success rate of 78% during an 18-month follow-up [15]. In this study, none of the nine individuals developed de-novo stress incontinence, and no significant bleeding was seen. These findings further support the feasibility of dorsal urethroplasty in selected patients with a history of unsuccessful urethral procedures, while acknowledging the limited size and follow-up of available series.

Another factor deemed significant as success in selecting a treatment is the likelihood of complications. The dorsal approach provides benefits, including enhanced vascularity attributed to the clitoral neurovascular structure on this side. This leads to effective graft sustenance and an elevated success rate. However, the rich vascular network also carries a risk of bleeding. Although theoretically the risk of bleeding is high, studies have shown that the rates of serious bleeding are negligible. Furthermore, the dorsal location of the female external urethral sphincter increases the risk of de novo stress incontinence after this operation [16]. Although de novo stress incontinence has been reported quite rarely in recent series, this risk is theoretically possible [4,6,8,11]. It is likely higher in cases of incorrect dissection planning or dorsal dissections extending to the bladder neck in long strictures. In our study, only one patient experienced mild stress incontinence, and the proximal location of the stricture in this patient supports this hypothesis. Given that the ventral vaginal bed remains intact in the dorsal onlay urethroplasty, the risk of urethrovaginal fistula is minimal. Additionally, this area remains natural for the possible future operations like mid-urethral slings.

Drug-coated balloon dilation has also been explored as a minimally invasive option for FUS, but current evidence is limited to a case report and a small series; therefore, its role relative to reconstructive urethroplasty remains to be defined [17,18].

The current study has several limitations. In addition to its retrospective nature, the median follow-up was only 12 months, which limits the assessment of long-term durability, particularly with respect to recurrence. Furthermore, a validated disease-specific patient-reported symptom or outcome measure was not prospectively administered. Consequently, postoperative symptoms were assessed clinically and their severity could not be quantified using a standardized instrument, which may have introduced subjectivity into the identification of symptomatic recurrence. Another limitation of the current study is that validated forms were not used to evaluate sexual function or quality of life metrics. Furthermore, objective functional measurements, including Qmax and PVR, were not systematically available beyond the 3-month assessment; therefore, the durability of the objective functional improvement could not be evaluated longitudinally. Later outcomes were primarily based on clinical recurrence and the need for additional intervention. As this series represented the surgeons’ initial experience with female DO-BMGU, the limited sample size precluded a formal assessment of the procedure-specific learning curve. In addition, smoking status was unavailable for 45% of patients, limiting the interpretation of this variable.

## 5. Conclusions

DO-BMGU was associated with early improvement in objective voiding parameters and a high rate of freedom from reintervention for symptomatic recurrence during a median clinical follow-up of 12 months. No major complications occurred. Given the retrospective design, small sample size, and relatively short follow-up, these descriptive findings should be interpreted as preliminary. Larger multicenter prospective studies with longer follow-up, systematic longitudinal objective testing, and patient-reported outcome measures are required to establish the long-term durability and functional benefits of this technique.

## Figures and Tables

**Table 1:** Baseline Characteristics of Patients.

	All Patients (*n* = 20)
Age, Mean ± SD	52.9 ± 14.7
BMI, Mean ± SD	28.1 ± 5.0
ASA score, *n* (%) ASA-1 ASA-2 ASA-3	4 (20%) 12 (60%) 4 (20%)
Smoking status, *n* (%) Never smoker Ex smoker Active smoker NA	9 (45%) 0 2 (10%) 9 (45%)
Etiology, *n* (%) Iatrogenic Idiopathic Infectious/Inflammatory Lichen sclerosus Traumatic	6 (30%) 11 (55%) 1 (5%) 1 (5%) 1 (5%)
Stricture location, *n* (%) Distal Midurethral Proximal Panurethral	7 (35%) 9 (45%) 2 (10%) 2 (10%)
Stricture length, cm, Median (IQR)	2 (2–3)
Previous urethrotomy, *n* (%)	3 (15%)
Previous dilatation, *n* (%)	20 (100%)
Number of dilatations, Median (IQR)	2.5 (1.8–4.0)
Preoperative UFM, Qmax mL/s, Mean ± SD	6.1 ± 2.3
Preoperative PVR, mL Median (IQR)	125 (94.5–155)

IQR: Interquartile range; PVR: Postvoid residual; Qmax: Maximum flow rate; ASA: American Society of Anesthesiologists; UFM: Uroflowmetry; NA: Not available.

**Table 2:** Perioperative Outcomes.

	All Patients (*n* = 20)
Operation time (minutes), Median (IQR)	65 (60–80)
Hospital stay (day), Median (IQR)	3 (3–4)
Foley catheter removal day, Median (IQR)	21 (21–21)
PO 3rd month Qmax mL/s, Mean ± SD	20.4 ± 5.8
PO 3rd PVR, mL, Median (IQR)	10 (10–40)
Follow up time (months), Median (IQR; range)	12 (12–24; 6–60)
Freedom from reintervention for symptomatic recurrence, *n* (%; 95% CI)	18 (90%; 68.3–98.8)

IQR: interquartile range; PVR: postvoid residual volume; PO: postoperative; Qmax: maximum urinary flow rate; SD: standard deviation; CI: confidence interval.

**Table 3:** Postoperative complications and donor site morbidity.

**Postoperative Complications**	***n* (%)**
Bleeding requiring transfusion, n (%)	0
Vaginal/perineal hemetoma, n (%)	0
Clavien–Dindo grade II	4 (20%)
Postoperative urinary tract infection, n (%)	4 (20%)
Postoperative wound infection, n (%)	1 (5%)
Clavien–Dindo grade ≥ III	0
Fistula, n (%)	0
De-novo stress urinary incontinence, n (%)	1 (5%)
**Donor Site Morbidity**	***n* (%)**
Bleeding	1 (5%)
Oral donor-site inflammatory changes, n (%)	2 (10%)
Cheek swelling lasting > 3 days	1 (5%)
Hypergranulation tissue	1 (5%)
Difficulty with mouth opening and chewing lasting > 10 days	3 (15%)
Articulation difficulty lasting > 10 days	2 (10%)

## Data Availability

The data sets generated during and/or analyzed during the current study are not publicly available. The data are available from the corresponding authors on reasonable request.

## Abbreviations

The following abbreviations are used in this manuscript:

BMG	Buccal mucosal graft
BOO	Bladder outlet obstruction
DO	Dorsal onlay
FUS	Female Urethral Stricture
EAU	European Association of Urology
QMAX	Maximum urinary flow rate
UFM	Uroflowmetry
